# State of Knowledge of Coastal and Marine Biodiversity of Indian Ocean Countries

**DOI:** 10.1371/journal.pone.0014613

**Published:** 2011-01-31

**Authors:** Mohideen Wafar, Krishnamurthy Venkataraman, Baban Ingole, Syed Ajmal Khan, Ponnapakkam LokaBharathi

**Affiliations:** 1 Biological Oceanography Division, National Institute of Oceanography, Dona Paula, Goa, India; 2 Marine Biology Regional Centre, Zoological Survey of India, Chennai, Tamil Nadu, India; 3 Faculty of Marine Sciences, Centre of Advanced Study in Marine Biology, Parangipettai, Tamil Nadu, India; Lund University, Sweden

The Indian Ocean (IO) extends over 30% of the global ocean area and is rimmed by 36 littoral and 11 hinterland nations sustaining about 30% of the world's population. The landlocked character of the ocean along its northern boundary and the resultant seasonally reversing wind and sea surface circulation patterns are features unique to the IO. The IO also accounts for 30% of the global coral reef cover, 40,000 km^2^ of mangroves, some of the world's largest estuaries, and 9 large marine ecosystems. Numerous expeditions and institutional efforts in the last two centuries have contributed greatly to our knowledge of coastal and marine biodiversity within the IO. The current inventory, as seen from the Ocean Biogeographic Information System, stands at 34,989 species, but the status of knowledge is not uniform among countries. Lack of human, institutional, and technical capabilities in some IO countries is the main cause for the heterogeneous level of growth in our understanding of the biodiversity of the IO. The gaps in knowledge extend to several smaller taxa and to large parts of the shelf and deep-sea ecosystems, including seamounts. Habitat loss, uncontrolled developmental activities in the coastal zone, overextraction of resources, and coastal pollution are serious constraints on maintenance of highly diverse biota, especially in countries like those of the IO, where environmental regulations are weak.

## Introduction

**Figure 1:**
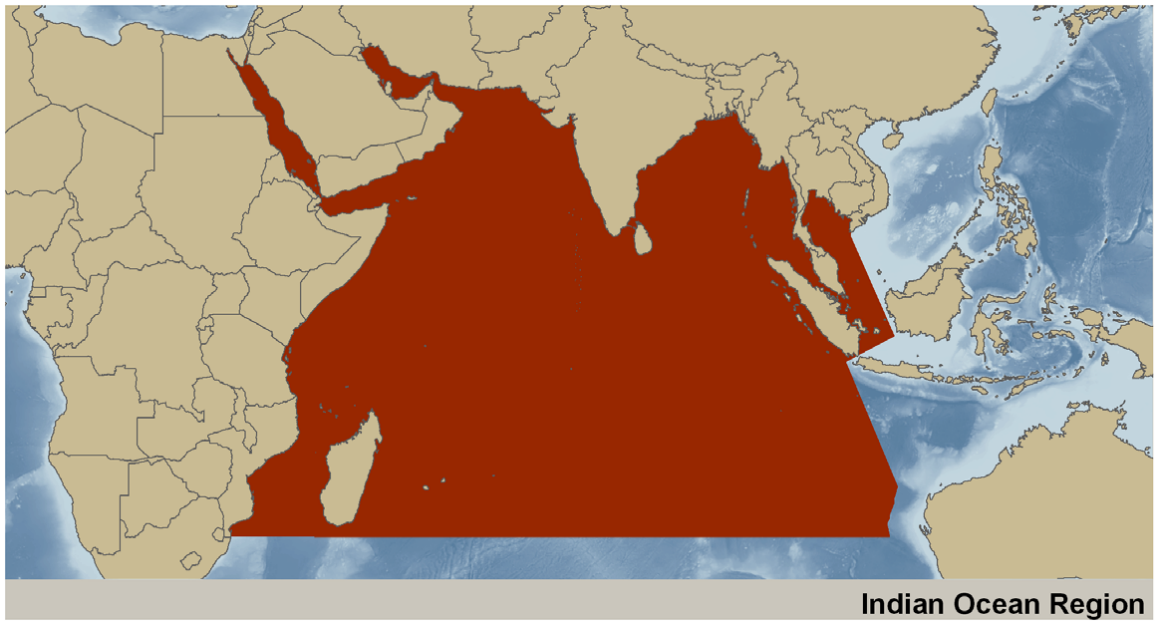
Geographical spread of the Indian Ocean. This map depicts the geographical limits of the IO as considered in this article for evaluation of the current state of knowledge of coastal and marine biodiversity.

The Indian Ocean (henceforth IO) is designated conventionally as an area between 25° N and 40° S and between 45° E and 115° E [1]. Meridionally, the IO extends from the Gulf of Oman and the head of the Bay of Bengal in the north to 40° S and zonally, from the east and South African coasts in the west to the coastlines of Myanmar, Thailand, Malaysia, and Western Australia in the east (Figure 1). The IO spreads over 74.92 million km^2^ (29% of the global ocean area) with an average depth of 3,873 m and a maximum depth of 7,125 m (Java Trench). The IO can be divided into two regions, the northern part comprising regional seas (Red Sea, Persian Gulf, Arabian Sea, and Bay of Bengal), and the southern, oceanic part, merging with the Southern Ocean. Water exchange between the IO and the Atlantic Ocean occurs around the southern tip of Africa and between the IO and the Pacific Ocean, through the Indo-Pacific through-flow between northern Australia and Java.

Several characteristics distinguish the IO from other oceans. The foremost is that it is landlocked to the north and the resultant differential heating of the landmass and the sea gives rise to a wind circulation that reverses direction, and entrains a corresponding reversal in surface circulation, twice a year. This monsoon effect has a significant bearing on climatology of the northern IO, in turn affecting the biological productivity and agrarian economy of the regional countries. The 36 littoral and 11 hinterland nations, all of which are regarded as developing countries, on the rim of the IO account for 30% of the world's population. The IO is also a significant contributor to the productivity of living marine resources, with estimated annual yields of 8 million tons of capture fisheries and 23 million tons of culture fisheries, equivalent, respectively, to 10% and 90% of the world's production [2]. The tropical nature of most of the IO countries also renders them sites of high coastal and marine biological diversity—for example, 30% of global coral reef cover (185,000–200,000 km^2^) [2], [3] lies in the IO region. The high population density of most countries is also a major cause of degradation of coastal habitats, especially through addition of pollutants. It has been estimated [4] that Indian coastal seas have been receiving 3.9 * 10^12^ liters of domestic sewage and 3.9 * 10^11^ liters of industrial sewage (taken as 10% of the former) every year. Such assessments are not readily available for all IO countries. Hence an extrapolation, using the ratio of the length of the coastline of India (6,500 km) to that of all IO countries (66,526 km) [3], would suggest that a pollution load of 40 * 10^12^ and 4 * 10^12^ liters, respectively, of sewage and industrial effluents may enter IO coastal seas every year. The consequences of this level of pollution, and the uncontrolled physical changes happening in the coastal habitats of all nations, seriously constrain the sustenance of biodiversity.

## Materials and Methods

### Major features of hydrology of the IO

Two noteworthy features in the hydrology of the IO have an influence on the distribution of biodiversity and productivity [5]. The first is the anomalous distribution of annual mean precipitation between the west (10 cm per year on the Arabian coast) and east (more than 300 cm per year near Sumatra and the Andaman Sea). This wide distribution has an impact on the surface salinity of practically the whole Bay of Bengal, which is fresher in the top few tens of meters and entrains a halocline. The second is the seasonal reversal of currents in response to changes in monsoon seasons from southwest to northeast and vice versa. This reversal is not purely wind driven, but occurs down to depths greater than 750 m. This reversal entrains seasonally variable rates of advection of nutrient-rich subsurface waters into the surface layers of the Arabian Sea and Bay of Bengal. As a result, at times there can be high biological productivity and bloom formations.

### Marine ecosystems of the IO

Open ocean waters cover more than 80% of the surface area of the IO (Table 1). Upwelling zones constitute a significant fraction of the coastal waters [6], [7]. The coral reef ecosystems spread over approximately 0.2 million km^2^ [3] and the mangroves extend over 40,000 km^2^ [8]. The sandy and rocky beach ecosystems, taken as a product of the length of the coastline of all maritime states (66,000 km) and an average intertidal zone width of 50 m, account for about 3,000 km^2^. The IO countries also have 246 estuaries each draining hinterlands greater than 2,000 km^2^ and a large number of minor estuaries, besides coastal lagoons and backwaters. The combined area occupied by the estuaries is not known, but it is worth mentioning that the Hooghly estuarine system in India (downstream region of the river Ganga) is one of the largest in the world and, along with the Brahmaputra estuarine region in Bangladesh, sustains the largest mangrove forest (the Sunderbans) in the world [9]. Another ecosystem worth mentioning is the hypersaline salterns [10]. These man-made ecosystems are localized to certain dry and arid regions in India, and their combined area may be between 5,000 and 10,000 km^2^. Their importance to the biodiversity of the region relates to the presence of salt-tolerant species such as *Artemia salina*, *Dunaliella salina*, and cyanobacteria, besides their role as home to resident and migratory avifauna. The IO is also home to 9 large marine ecosystems (LMEs) [11]. These include, from west to east, Agulhas Current, Somali Coastal Current, Red Sea, Arabian Sea, Bay of Bengal, Gulf of Thailand, West Central Australian Shelf, Northwest Australian Shelf and Southwest Australian Shelf.

**Table 1:** Areal spread of marine ecosystems in the Indian Ocean.

Ecosystem	Area (in million km^2^)
**Open ocean**	
Oligotrophic	19.6
Transitional areas	23.8
Equatorial divergence	18.9
**Coastal**	
Upwelling zones	7.9
Other neritic waters	5.3
**Other**	
Coral reefs	0.2
Mangroves	0.04
Sandy and rocky beaches*	0.004
Estuaries	—
Hypersaline water bodies/lagoons	<0.005

*length of coastline multiplied by an average intertidal width of 50 m.

References: [1], [3], [6]–[8].

### History of marine biodiversity research

The evolution of marine biological research in the IO region is partially linked to the colonial past of many countries, the International Indian Ocean Expedition, and modern-day programs. In most of the countries during pre-independence era, as in India and Sri Lanka and some island nations, collection and cataloging was done almost exclusively by European scientists, and the specimens and data were archived in museums abroad. The importance of their work cannot be minimized nonetheless. The two-volume publication in 1878 on the fishes of India by Francis Day, among others, is a classic example that is referred to even today. To this should be added the numerous memoirs, monographs, and expedition reports, such as those of *Challenger* (1872–76), *Investigator* (1801–03), and *Dana* (1928–30). Appendix S1 provides a list of important treatises on many marine taxa that serve as taxonomic guides for the fauna and flora of the Indian Ocean.

While coastal and marine biodiversity (CMB) studies in the IO region during the nineteenth century and early part of the twentieth century were mostly on those specimens from neritic waters and physically accessible habitats, the International Indian Ocean Expedition (IIOE, 1960–65) enabled sampling of the full extent of the IO by 40 research ships, with logistic support and participation from 20 countries, including some outside the region [12]. Besides considerably enhancing taxonomic knowledge of the open-ocean species, mainly zooplankton, the IIOE is also distinguished in two other respects. First, it enabled collection and use of oceanographic parameters to explain the abundance and distribution of planktonic species and their productivity in the IO region. Second, it laid the foundation for modern-day research on marine biological diversity in most of the regional countries, both institutionally and in manpower generation, especially in India.

Research on marine biological diversity in the current phase (last five to six decades) in the region is distinguishable by three traits. The first is the desire by most countries to develop national capacity through manpower and institutional strength, a process aided by international, regional and bilateral training, and collaborative programs. The second is the awareness of the countries of the need to address biodiversity changes in response to anthropogenic forces (and to some extent natural forces) prevailing locally. The third, and perhaps the more serious, is the vast imbalance in capacity among the nations. For example, among the IO countries, India is notable for the large number of oceangoing research vessels, large scientific and technical manpower, capability for using advanced technologies (for example, remote sensing, DNA fingerprints), and the capacity for exploring deep seas and the southern part of the Indian Ocean, extending to the Antarctic continent. This imbalance, even among countries other than India, has a telling effect on our understanding of the biological diversity in the region. One only needs to compare the data (or rather the paucity of data) between adjacent countries like Kenya and Somalia (1813 and 147 citations respectively in Aquatic Science and Fisheries Abstracts and Indian Ocean bibliographic database of the National Institute of Oceanography, Goa for Kenya as against 242 and 26 for Somalia) or Malaysia (2318 and 58 citations) and Myanmar (183 and 37 citations) to appreciate this.

## Results and Discussion

### Current status of coastal and marine biodiversity in the IO

The Ocean Biogeographic Information System (OBIS – www.iobis.org) provides the following species abundance data for the Indian Ocean: animalia, 30894: archaea, 4; bacteria, 864; chromista, 773; fungi, 75; plantae, 1690; protozoa, 689, totaling 34,989 species. For further analysis of the pattern of distribution of CMB among the IO countries, we used data from those papers presented in an international workshop on coastal and marine biodiversity [3], [13]–[19], species listing in the Marine Species Database for Eastern Africa (MASDEA) [20], and unpublished manuscripts by Osore and Fondo for Kenya, Bijoux for Seychelles, and Rabanavanana for Madagascar. Because of the large range of habitats, from estuaries to abyssal plains, we organized this review of CMB under three thematic regions: continental nations, island nations, and deep-sea ecosystems. Australia and South Africa are not included, as they are dealt with separately elsewhere [21], [22] in this collection.

#### Continental nations

Among the continental nations, the most comprehensive account of CMB is that from India, which reports 15,042 marine species (Table 2). Practically every taxon of the plant and animal kingdoms has been investigated, though the numbers reported may underestimate those actually occurring. Some exceptions, however, should be noted. For example, the inventory of 844 macroalgae for India [23] would most likely be close to complete, given the extensive sampling and geographical limits of the macroalgal habitats. The highest diversity reported, besides that from India, comes from Indonesia with 10,855 species. Some countries from which biodiversity inventories are difficult to access in the recent years, such as Myanmar, nevertheless have good records of diversity of some groups, as is evident from the 310 species of plankton recorded by Win [24]. Griffiths [14], synthesizing the data for the East African countries and island nations, arrived at a marine species count of 11,257 for the western IO. He [14] also noted that no reliable or comprehensive lists exist for individual nations and that the existing taxonomic coverage is conspicuous in omission of small organisms.

**Table 2:** Marine species diversity known from some Indian Ocean countries, listed by major taxa.

Taxon	India	Malaysia	Kenya	Madagascar	La Réunion	Indonesia	Seychelles
PLANTAE							
Diatoms	200+	70					
Dinoflagellates	90+	30					
Macroalgae	844	196	176	108	179	782	316
Seagrasses	14	14		12	2	13	8
Mangroves	39	104	9			38	8
PROTISTA							
Protozoa	532+						
Foraminifera	500+						
Tintinnids	32+						
ANIMALIA							
Porifera	486+	10	2	306	19	830	351
Cnidaria	842+		183	435	459	1150	>300
Ctenophora	12+						
Platyhelmintha	350						
Annelida	338	61	10		75		5
Chaetognatha	30+	10					1
Sipuncula	35	5					
Echiura	33						
Gastrotrocha	75						
Kinorhyncha	10						
Tardigrada	10+						
Crustacea	3498	1245	343	779	192	1512	75
Mollusca	3370	430	297	1158	2500	2500	200
Bryozoa	200+			99			
Echinodermata	765	88	93	227	61	747	150
Hemichordata	12			182			
Protochordata	119+						
Pisces	2546	1500	662	739+	858	3215	>1000
Reptilia	35	40	3	5	4	38	4
Mammalia	25	29	25	10	3	30	26
Total	15042	3832	1803	4060	4352	10855	2444

Published information on the marine biodiversity of the countries of the Red Sea and Persian Gulf region is generally biased toward larger groups, while groups such as sponges, ctenophores, octocorals, polychaetes, and tunicates are poorly known [25]. Again, most of what we know of species diversity of the region comes from coral reef ecosystems, especially of the Red Sea [26], [27]. However, using the relationship between the number of species of echinoderms recorded by Richmond [26] and the potential number of all marine species (see below), it is possible to arrive at a potential species count of about 5,400 for the Red Sea region.

#### Island nations

The IO islands range geographically from oceanic to continental and physiographically from low to high, but they are geologically varied, including volcanic, limestone, granite, metamorphic, and mixed types [28]. The complete list of all IO islands, their location, geomorphology, coastal ecology, and disturbances to biodiversity can be accessed from the synthesis by Wafar et al. [28]. This synthesis reveals two important characteristics of CMB. The first is the poor coverage in the inventory of CMB of all islands, and the second is that where such data are available, they are usually related to corals, fishes, and mollusks. Thus, while we have a reasonably good estimate of the number of coral species in all these island nations, data on other groups are virtually nonexistent.

Some large island nations, however, appear to be exceptions where inventories for the diversity of more than one group (and often the coral, fish, and mollusk combination) are available (Table 2).

Review of the available literature on CMB of IO countries thus showed that

1. Among all IO countries, only India has a relatively more comprehensive marine biodiversity database.
2. Data from countries like Somalia, Myanmar, and all Gulf nations bordering the Red Sea and Persian Gulf are scarce, and are limited to some species or groups.
3. The only group that has been well catalogued across the region appears to be the fishes, obviously because of their economic importance.
4. Corals and mollusks rank next in importance and quality of data.
5. The CMB databases of some countries are heavily biased toward some groups, such as the 830 species of poriferans inventoried from Indonesia, obviously because research efforts (e.g., Siboga and Snellius II expeditions) made for some groups were more intensive than those made for other groups.

Given that published synthetic accounts of CMB for most countries of the IO are either nonexistent or extremely difficult to come by, we adopted a statistical (regression fits) approach to “estimate” the number of marine species in the IO countries. For this, the data from India, Malaysia, Kenya, Madagascar, Indonesia, Réunion, Seychelles (Table 2) and the Western Indian Ocean (WIO) [20] were considered because they cover more groups and are more detailed than those for other countries. Using these data, we derived linear relationships between the number of fish/mollusk/echinoderm species inventoried and the total number of species. The underlying assumption is that when the number of groups inventoried is large, as in the case of these countries and the WIO region, the species diversity of some groups could then become reliable proxies for the total species diversity. This, however, does not place any upper limit on the number of species discoverable for any one nation, since the inventories for the most surveyed groups like fishes, mollusks and echinoderms could still be underestimates.

**Figure 2:**
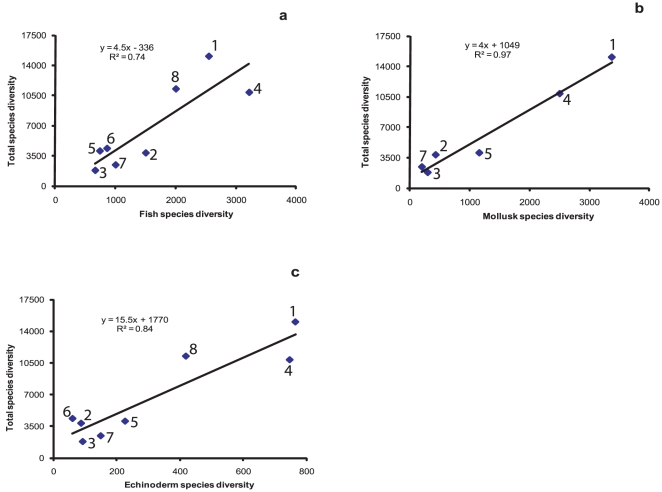
Relationships between (a) fish, (b) mollusk, and (c) echinoderm diversity and total species diversity. These relationships have been obtained by fitting linear regressions of total marine species diversity on fish, mollusk and echinoderm species diversity as known from sources considered in this article. These include India (1), Malaysia (2), Kenya (3), Indonesia (4), Madagascar (5), Réunion (6), Seychelles (7) and Western Indian Ocean (8).

**Figure 3:**
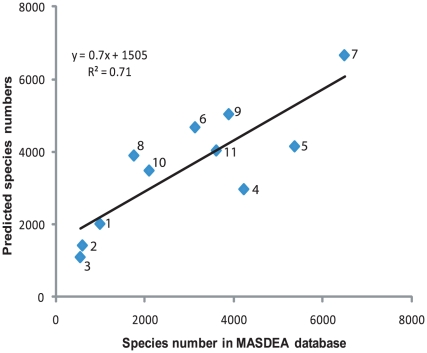
Relationship between species numbers in the MASDEA database and those predicted from fish diversity. The statistically significant relationship obtained between known fish species and total species diversity from some IO countries has been used to predict potential species numbers for IO counties. Values predicted for some East African countries and the island nations in the western IO were then compared with the data as known from the MASDEA database for these countries. 1. Comores, 2. Djibouti, 3. Eritrea, 4.Kenya, 5.Madagascar, 6. Mauritius, 7. Mozambique, 8. Réunion, 9. Seychelles, 10. Somalia and 11. Tanzania.

The relationships between the species numbers in these three groups and the total diversity reported were indeed statistically valid (Figure 2) and provided a mechanism for indirectly assessing the total potential marine biodiversity of a given nation. We also analyzed the reliability of this relationship by comparing the predicted values with those reported in the MASDEA [20] database (Figure 3). The relationship, when tested with the data for 11 East African countries, was highly significant; the MASDEA database reported, on an average, only 23% less than the possible biodiversity.

Using the marine fish diversity as the independent variable, as this is probably the best inventoried in all the IO countries (www.fishbase.org), we estimated the CMB for all IO countries. Table 3 summarizes the diversity values collected from both published and unpublished sources, the MASDEA database and deduced from the relationship discussed above. The predicted biodiversity estimates could still be underestimates (e.g., values predicted for countries of the Persian Gulf), because they are based only on a current understanding of the size of fish and total marine species diversity in some countries. The potential diversity occurring in IO countries could be several times higher than these estimates, given that several smaller groups are usually neglected and spatial coverage is still poor. Again, the prediction is for only nearshore waters whereas the large oceanic area and hundreds of coral reefs, both of which are potential sites of new discoveries, still remain unsampled.

**Table 3:** Species diversity of Indian Ocean countries, as known from the MASDEA database, as reported during the Census workshop on coastal and marine biodiversity of the Indian Ocean, and as estimated from the relationship of fish species diversity to total diversity (Figure 2).

Country	Number of species		
	MASDEA	IO-CoML	Predicted
Bahrain			591
Bangladesh			1208
Chagos (BIOT)	1668		
Comores	995		2027
Djibouti	600		1432
East Timor			339
Egypt			3026
Eritrea	551		1109
India		13327	
Indonesia		10855	
Iran			1424
Iraq			294
Israel			2409
Jordan			2049
Kenya	4232	1452	
Kuwait			672
Madagascar	5374	3944	
Malaysia		4382	
Maldives			4700
Mauritius	3134		4686
Mozambique	6492		6671
Myanmar			1851
Oman			4083
Pakistan			1896
Qatar			393
Réunion	1758	4352	
Saudi Arabia			1761
Seychelles	3892	2887	
Singapore			1982
Somalia	2101		3494
Sri Lanka			3921
Sudan			1219
Tanzania	3612		4047
Thailand			6105
United Arab Emirates			483
Yemen			2868

Some patterns in the distribution of CMB can be recognized. The first is that the continental nations, straddling the tropical belt and having a larger diversity of habitats, have the largest CMB (India, Indonesia, Mozambique, Thailand, and Malaysia). Large island nations (e.g., Maldives, Réunion, Mauritius, Madagascar, and Sri Lanka) rank next in importance. These are followed by a group consisting of continental nations with less coastal area and less diversity of ecosystems, notably the absence of coral reefs (e.g., Pakistan, Bangladesh, Myanmar). Smaller island nations form the next category (for example, Singapore, Comoros). The nations adjoining the Persian Gulf (e.g., Qatar, UAE, Bahrain) have the lowest diversity. However, those Gulf region countries that have coastal areas in the Mediterranean (e.g., Egypt) or the Arabian Sea (Oman, Yemen) have relatively higher numbers of species.

The surprising similarity between the CMB of continental nations with large coastlines and the large island nations can perhaps be related to the presence of coral reefs. The Indian Ocean is home to about 30% of the world's coral reefs. Of the 793 coral species recorded worldwide, 719 occur in the Indo-west Pacific region [29], and when added to the diversity of other coral reef fauna (fishes, crustaceans, mollusks, polychaetes), the contribution of coral reefs could well be up to 20% or even more of the whole CMB of any country with luxuriant coral reefs. For example, the coral (about100 species) and reef fish (about 600 species) diversity of the Lakshadweep reefs alone accounts for 5% of the total marine species recorded from India.

In order to determine the extent of endemism, we analyzed the OBIS data, first by sorting records that are unique for each country in the IO region from among the full database, then pooling these together and removing all duplicate records. This gave a count of 8,795 species and, compared with the 34,989 species count for IO, represents an endemism of 25%. This is somewhat similar to the 28% known for South Africa and Australia [30] (both of which border IO and are either contiguous (South Africa) with, or not distant (Australia) from, other IO countries) but only half of that known for New Zealand (51%) and Antarctica (45%). A similar analysis for India (continental shores and Andaman and Nicobar Islands) gave a count of 2,372 species out of 15,042 known species, equivalent to about 16%, which is surprisingly similar to the average of 17% reported [30] across several NRIC regions. Endemism, however, is a relative expression, the magnitude of which is determined by the spatial scale of analysis and extent of sampling coverage. While geographically isolated regions like New Zealand and Antarctica may have high percentages of endemic species, their proportion decreases rapidly, down to around 10% or even less, in regions which are contiguous with the neighbouring ones [30].

#### Pelagic open ocean

The International Indian Ocean Expedition (IIOE) was the first attempt to describe quantitatively the geographic distribution and abundance of zooplankton in the Indian Ocean. During IIOE, 2,145 standard (using Indian Ocean Standard Net) zooplankton samples were collected in the 0–200 m depth range and sorted into various zooplankton groups. Besides enabling preparation of atlases of distribution of zooplankton biomass in the IO, the samples also were useful in preparing zooplankton biodiversity catalogues [31].

The groups that were intensively studied were copepods, ostracods, amphipods, chaetognaths, hydromedusae, cumaceans, euphausiids, and appendicularians, among others. Thanks to the IIOE collections, our knowledge of the zooplankton diversity of the open ocean has increased substantially. While it is difficult to summarize description of every group of zooplankton from IIOE, we could cite, as representative examples, the additions of the 21 species of chaetognaths [32] or the 46 species of siphonophores [33] to the oceanic zooplankton inventory. A comprehensive list of species described from IIOE samples is still under construction (www.CMarz.org), but it is safe to predict that it could be in the order of several hundreds.

#### Deep-sea habitats

Long sections of mid-ocean ridge divide the IO into a number of major basins. Some of these ridges are nonseismic, whereas others, like the Carlsberg, Mid-, Southwest, and Southeast Indian Ridges, are seismically active.

Until the 1970s, deep-sea benthos of the IO was collected only during major expeditions such as *Valdivia* [34], [35], *Albatross* [36], [37], *and Galathea* [37] and Soviet cruises [38], [39]. The Indian program on deep-sea benthos began as a part of surveys for polymetallic nodules in the Central Indian Ocean Basin (CIOB) in the 1980s. This continued through the 1990s (and is still continuing), as part of sea-bottom surveys for environmental impact assessment, prior to deep-sea mining. Parallel programs on the geology and geochemistry of mid-ocean ridges also provided opportunities to collect deep-sea fauna. As a result, a fairly comprehensive dataset on the type and distribution of deep-sea benthos has been generated and compiled recently [16].

#### Meiofauna

The meiofauna assemblages of the abyssal plains of the IO are made up of 20 metazoan groups (Nematoda, Turbellaria, Harpacticpoida, Gastrotricha, Foraminifera, Cumacea, Polychaeta, Kynorincha, crustacean nauplii, Tardigrada, decapod larvae, Zoea, Halacarida, Amphipoda, Tanaidacea, echinoderm larvae, Isopoda, Ostracoda, crinoid larvae, Nemertina, and Hydroida) [16]. Among these, the nematodes are numerically dominant, accounting for 37% of the density of meiofauna, followed by turbellarians (35%), gastrotriches (11%), polychaetes (9%), harpacticoid copepods (4%) and other minor groups (4%).

#### Macrofauna

The macrofauna of the CIOB comprises 24 major groups belonging to 15 phyla, among which species of Protozoa, Porifera, Mollusca, Annelida, Arthropoda, and Echinodermata are predominant (Table 4). Polychaeta is the dominant group in terms of number of individuals, contributing to 33% of the total macrofaunal community. Crustaceans (23%) formed the most diverse group, with species from 10 taxa: amphipods, isopods, ostracods, harpacticoides, Paracaridean shrimps, thalassinoid decapods, cumaceans, brachyuran crabs, pagurid crabs, and Tanaidaceans. Miscellaneous forms such as turbellarians, hydrozoans, sponges, sipunculid worms, siphonophores, and fish larvae account for about 7% of the faunal composition.

**Table 4:** Composition of the macrofauna in the Central Indian Ocean Basin and the dominance of major taxa in the known diversity, in decreasing order.

Taxon	Percent dominance
Polychaeta	32.9
Gastropoda	9.3
Amphipoda	7.1
Isopoda	5.3
Bivalvia	4.4
Unidentified groups	4.2
Ostracoda	4.0
Nematoda	4.0
Oligochaeta	3.9
Harpacticoida	3.8
Foraminifera	3.5
Echinoidea	1.7
Bryozoa	1.6
Nemertina	1.5
Echiuridae	1.5
Branchiopoda	1.2
Ophiuroidea	1.2
Radiolarian	1.1
Paracaridean shrimps	0.9
Turbellaria	0.9
Hydrozoa	0.8
Sipuncula	0.8
Thalassinoid Decapoda	0.8
Cumacea	0.6
Holothuroidea	0.6
Fish larvae	0.5
Dentaliidae	0.4
Brachyuran crab	0.3
Monoplacophora	0.2
Pagurid crab	0.2
Siphonophora	0.1
Soft coral	0.1
Sponges	0.1
Pteropoda	0.1
Tanaidacea	0.1

#### Megafauna

Ingole et al. (in prep.) investigated the composition and behavior of deep-sea megafaunal assemblages from deep-tow photographs (still and video) taken by Indian research vessels. They recognized 11 invertebrate groups (Xenophyophorea, Porifera, Hydrozoa, Pennatularia, Actiniaria, Ascidiacea, Crustacea, Holothuroidea, Echinoidea, Asteroidea, Ophiuroidea) and one vertebrate group (Osteichthyes) besides several unidentifiable forms. The echinoderms were represented by asteroids (*Hymenaster violaceus*), ophiuroids (*Ophiura* sp.), echinoids, and holothuroids (*Mesothuria murrayi*, *Molpadia* sp., *Pseudostichopus* sp.). The density of megafauna ranged from three to eight species per 100 m^2^ of the surface area photographed.

#### Seamounts

Oceanic seamounts are the least explored of all marine ecosystems in the IO. The biodiversity of the seamounts is so poorly known that even as of today the number of species recorded [40]–[46] remains less than 300 (Table 5). Dredging done on Afanasiy Nikitin seamount yielded few species of soft corals, sponges, and echinoderms (Ingole et al. in prep.). Ingole et al. (in prep.) also identified several nematodes (*Halalaius, Eumorpholaimus, Araeolaimus, Linhystera, Diplopeltula, Rhabditis, Paraethmolaimus, Sabatieria, Odentophora, Axonolaimus, Spiliphera*), harpacticoides (*Parameriopsis* sp.), and polychaetes (*Hesione* sp., *Prionospio* sp., *Paradoneis*) in the box core collections from Afanasiy Nikitin seamount and two other, as yet unnamed, seamounts.

**Table 5:** Diversity records for some seamounts in the Indian Ocean.

Name of seamount	Number of species							Reference
	Proto-zoa	Zoo-plankton	Fish	Mega-benthic	Macro-benthic	Meio-benthic	Endemic species	
Equator, Fred, and Farquhar	0	0	90		0	0	0	[38]
Walters Shoals	0	0	20		0	0	8 (fishes)	[39]
Equator	0	6	0		0	0	0	[40]
Error, Equator, Fred, Farquhar & off northwestern Madagascar	0			50 Cephalo-pods	0	0	0	[41]
Farquhar	8	0	0		0	0	0	[42]
Walters Seamount	0	7	0		1	1	0	[43]
Southwestern Indian Ridge seamounts	0	0	16		0	0	0	[44]
Mid-Indian Ridge and Broken Ridge	0	0	10		0	0	0	[44]
Afanasiy Nikitin				20				Ingole (unpub.)
Unknown				0	6	8	0	Ingole (unpub.)

#### Mangrove ecosystems

Another habitat in the IO for which comprehensive biodiversity information is available is the mangrove ecosystem. Spalding et al. [47] assembled data from Indo-Malaysian and Australasia mangroves of the IO and reported 1,511 and 754 faunal species, respectively. A substantial fraction of this was, however, in the form of associated fauna such as insects, birds, and nonmarine mammals. Kathiresan and Rajendran [17] provided a comprehensive account of fauna and flora from Indian mangroves (Table 6). The total number (3,959) is astonishingly high and even after omitting insects, birds, and (possibly non-marine) mammals, the number would be in the region of 3,000. A good fraction of this is related to microbial diversity, which is not generally reported or included in inventories.

**Table 6:** Total number of floral and faunal species in mangrove ecosystems of India.

Taxon	Number of species
I. Mangroves	39
II. Mangrove-associated flora	
Bacteria	69
Fungi	103
Algae	559
Lichens	32
Actinomycetes	23
Seagrasses	11
Salt marsh vegetation and other halophytes	12
III. Mangrove-inhabiting fauna	
Crabs	138
Prawns	55
Mollusks	308
Other invertebrates	745
Insects	711
Fish	546
Fish parasites	4
Birds	433
Amphibians	13
Reptiles	85
Mammals	70
Total	3959

#### Microbial diversity

India is a member of the International Census of Marine Microbes, a project of the Census of Marine Life (Census), and has been able to apply 454 tag sequencing technology to select samples from mangrove, beach, continental slope, seamounts, and deep-sea ecosystems [48].The results of these surveys include enumeration of 44 different phyla in mangroves and 35 in beach sediments; high abundance (83% to 88%) of Proteobacteria of the microbial community in the continental shelf, seamount, and CIOB sediments; and near-total dominance by *Erythrobacter*, an Alpha-proteobacterial member of the aerobic anoxygenic phototrophic lineage that has been detected in many hydrothermal and oligotrophic systems, of the bacterial community in pelagic red clay sediments of the Central Indian Ocean Basin.

Microbial surveys, done as part of the Census project Biogeography of Deep-Water Chemosynthetic Ecosystems, showed relatively higher abundance (up to 10^5^ CFU (colony forming units)L^−1^) of nitrifiers and manganese- and cobalt-tolerant bacteria, synchronizing with relatively high methane and metal concentrations at potential locations along the Carlsberg and North Central Indian Ridges [49]. What is more interesting is the likelihood that the deep ocean oligotrophic sediments have retained their chemosynthetic potential: at 1 atm (0.1013 MPa) and at 5°C, the siliceous oozes of CIOB fixed 5–45 nmol C (g dry wt)^−1^ d^−1^ and red clay with volcanic signatures fixed 230–9,401 nmol C (g dry wt)^−1^ d^−1^ [50]. The rate of carbon fixation in CIOB sediments thus is comparable to that in white smoker waters and one to four orders of magnitude less than that of bacterial mats and active vents.

### Known, unknown, and unknowable

Allowing for some overestimation (the 104 mangrove species reported [19] for Malaysia possibly include species other than true mangroves) or approximation (the number of mollusks reported for both La Réunion and Indonesia is 2,500 and is probably a rounded-up figure), the current inventory of CMB in the IO region, as known from OBIS data, stands at about 35,000 species.

**Figure 4:**
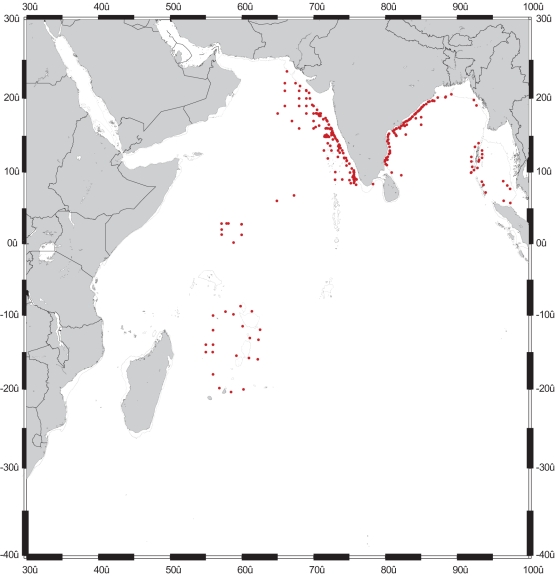
Distribution of stations for sampling benthos in the Indian Ocean. The station positions given in this map are, largely, those occupied by Indian research vessels, RV *Gaveshani* and ORV *Sagar Kanya.*

Several constraints are evident in the use of existing data to estimate the true CMB of the IO. The first is the scarcity of data on many taxa of little or no economic interest at present. Table 2 shows that even for the countries where CMB records are available, existing data do not cover more than a quarter of the groups recorded from India. Second, when the animal and plant groups are compared, it becomes evident that the paucity of information is more acute among animals than plants, reflecting also the greater diversity in animal groups. Third, the inadequacy of the spatial coverage stands out clearly in most instances. Not only do several countries have little spatial data, but even within countries like India, which are reasonably well surveyed, the gaps are obvious. For example, among the 200 or so estuaries on the two coasts of India, only few major ones have been surveyed for biodiversity. Similarly, what we know of coral diversity in the Andaman Islands comes only from collections in the Wandoor National Park and the few islands surrounding it, whereas the total number of the islands in the Andaman-Nicobar chain exceeds 500. Likewise, most of the collections on deep-sea fauna are limited to the CIOB and a few seamounts from other basins (Figure 4). The poor knowledge of biodiversity of seamounts is particularly of concern and has profound implications for conservation. Because of the smaller size of the seamounts and considerable distances between them, many taxa tend to be localized in their distribution. Consequently, these taxa are highly vulnerable to the impacts of fishing and to possible extinction if recruitment from other seamounts does not happen.

The gap on temporal scales is also of concern. Discrete, one-time sampling may not only fail to record some seasonally occurring forms, but also give little information about changes in the species composition, including local extinction, over time as a consequence of natural events or human interference.

### Constraints on sustainability of coastal and marine biodiversity of the IO

Anthropogenic drivers affecting coastal habitats is a known phenomenon and a global map of human impacts on marine ecosystems produced by Halpern et al. [51] shows that no area of the ocean is unaffected and a large fraction (41%) is affected by multiple anthropogenic drivers, with large areas of high predicted impact occurring in some coastal ecosystems, some of them with high population densities.

In the context of IO, habitat loss is an important threat to the sustenance of CMB. Nowhere is the impact of habitat loss more evident than it is for mangroves. The systematic refrain from most IO countries is that mangroves are being cut down to make way for buildings, roads, and aquaculture farms [8]. In Malaysia, removal of mangroves has led to a phenomenal weakening of their role in coastal protection and as nurseries for larval and juvenile forms [18]. In Indonesia, a decadal loss of mangrove cover to brackish-water shrimp and fish farms was estimated to be about half a million hectares in early 1990s [15].

Loss of biodiversity through habitat loss is difficult to quantify in several other coastal habitats, because these impacts tend to be localized. For example in Goa (India), the loss of sand dunes and associated flora is near total because of ill-conceived beach beautification schemes and reclamation of sandy beach areas for recreational activities associated with tourism.

Pollution is another serious threat to sustenance of CMB, and the pollutants enter coastal waters mainly in two forms – as nutrients from domestic sewage and agricultural runoff and as industrial effluents (see above). Excess nutrients cause eutrophication and attendant hypoxia, killing the local fauna and flora. Industrial pollutants act directly as toxic substances, causing impairment in metabolic functions and eventually mortality. Dead zones in the coastal waters caused by eutrophication have been exponentially increasing since 1960, and of about 400 such zones catalogued recently [52], about 10 exist in the IO region. Though the problem does not appear to be as serious as it is in Europe, for example, the potential for a greater number of dead zones in the IO is indeed high, given that systematic data for many such polluted regions are rarely acquired in developing nations.

Overharvesting of exploitable marine biological resources can cause a decline in the stock locally and regionally [53], but to our knowledge, there has been no record of total extinction of a species because of overharvesting anywhere in the IO region. However, overexploitation of pelagic and demersal stocks at regional levels has been a recorded phenomenon at least since the 1980s [54]. To this should be added increased subsistence exploitation of resources, especially from reefs, mangroves, and estuaries. Another serious concern is the lack of enforcement of fishery regulations: nonadherence to mesh size regulation, trawling in estuarine waters, disregard of closed seasons and areas closed for fishing, and failure to use turtle excluder devices are some examples. In a discussion on management effectiveness of the world's marine fisheries Mora et al [55] concluded that conversion of scientific advice into policy, through a participatory and transparent process, is at the core of achieving fisheries sustainability, regardless of other attributes of the fisheries. In India, willingness by fishermen to adhere to closure of fishing in monsoon months during the last few years has reduced pressure on spawning populations. However, benefits of this closure are offset by intense uncontrolled fishing efforts once the season opens. This is because no acceptable MSY estimates for local fisheries exist.

Natural causes affecting CMB have also been of concern in recent years. These effects occur mainly in the form of shoreline changes due to rising sea level and physiological impairments, such as bleaching in corals, related to high sea surface temperature. The extent of the loss of species diversity resulting from these causes, however, is not known.

### Issues for the future

The foremost issue in improving the state of knowledge of coastal and marine biodiversity in IO countries is strengthening the taxonomic capacity base throughout the region. It has been variously hypothesized, from comparisons of the known diversity of a certain group in a region to the total number known in that group from other intensively studied regions or its global abundance, or from projections of the abundance of number of species in a smaller sampled area to the total area of the habitat in the region, that described biodiversity is only a fraction of what remains to be discovered. In light of the dwindling population of taxonomists, however, the magnitude of the task ahead is obvious. In India alone, all the scientists specialized in zooplankton taxonomy during and after IIOE and those of the Central Marine Fisheries Research Institute who have developed expertise in diverse phyla are no longer in active service, nor have they left any legatees. Presumably, a similar situation exists in IO countries that have even less budgetary provision for generation of taxonomist positions, or for support of full-time taxonomic research. Even among the younger generation of marine biologists, taxonomic research has practically no attraction as a career in relation to careers in fields such as marine biotechnology or aquaculture. The argument that without taxonomic expertise, marine biology becomes a dead subject does not bring students to taxonomy because the argument does not remove the tedium of research in systematics. Moreover, following the classical approach to taxonomy, one does not become an expert before completing a decade of intensive studies.

Two options are possible to resolve this impasse. The first is to make taxonomy an easier subject to master through the use of tools such as computer-aided taxonomy, pattern recognition, image analysis, and DNA fingerprinting. The second is value addition to taxonomic research, such as the need to have a correct taxonomic identity established in dealing with extraction of bio-products, genetic manipulation to enhance product yield, and biosafety. One other area where taxonomy can be made attractive is sustained monitoring of coastal ecosystems for natural and man-made changes. This requires, besides measurements of routine water quality parameters, also data on biological diversity, which often is a harbinger of changes to come. Contrasted with descriptive taxonomy, these approaches come with incentives of job security and future prospects that could make taxonomy more attractive as a career.

The second issue of concern is the existence of gaps in the spatial and temporal coverage of CMB inventories. Given the vast difference between the number of species known now and those waiting to be discovered, increased spatial coverage is critical. Some obvious areas where gaps exist are continental shelves and deep seas, including seamounts. Even along the 60,000 km coastline of IO countries, there are vast stretches that have never been sampled. Similarly, we also need to increase the frequency of temporal coverage, which could tell us whether a given species still exists at a given site. Closing gaps in both temporal and spatial coverage depends on availability of ship time and increased budgetary provision for census-type work. In this context, it is crucial to increase awareness among governmental funding agencies, international organizations, and donor agencies of the need to support biodiversity research.

### Implementation of Census of Marine Life initiatives

The Census of Marine Life is an international program designed to assess and explain the changes in the biodiversity of the world oceans, using a variety of tools ranging from classical surveys to technologies such as 454 tag sequencing strategy for microbial biodiversity. Besides creating outputs of excellence and immediate utility (such as the Ocean Biogeographic Information System), the Census has been striving to ensure its legacy. The Indian Ocean chapter of the Census (IO-CoML) came into existence in December 2003, with the organization of the workshop on coastal and marine biodiversity of the Indian Ocean. Since then the IO-CoML has been in the forefront to promote research on marine biodiversity in India and the region.

Of more than 40 new species described from the IO region in the last few years, 10 (6 mysids, 2 chaetognaths, 1 littoral mite, and 1 deep-sea sponge) were discovered by scientists working within the region. IO-CoML has also enabled implementation of barcode of marine life, with two training programs, one brainstorming session, establishment of the barcoder's network, and securing funds for a new project on barcoding of marine life from the Lakshadweep Islands. The Census nearshore project, NaGISA, began in the IO region with a training course organized by the co-ordinating agency, the Kenya Marine Fisheries Research Institute, in 2006 wherein the first set of sampling stations were established in a rocky beach and a seagrass bed near Mombasa, Kenya. Subsequently, the National Institute of Oceanography in India established NaGISA stations (rocky shore near Goa and seagrass bed in a Lakshadweep atoll) and began regular sampling. As part of the Census of continental margins project, COMARGE, two deep-sea and three coastal cruises on Indian and German research vessels were undertaken and the species-level data on nematodes and polychaetes have already been shared with the COMARGE database. Scientific cruises organized by IUCN and its partners on the seamounts of the SW Indian Ocean led to the collection of more than 7000 samples, analysis of which led to records of several species new to the region (http://seamounts 2009.blogspot.com) and several others with a potential of being new to science. The portal IndOBIS (www.indobis.org) has nearly 45,000 records now. The site is now being hosted from the Centre for Marine Living Resources and Ecology (Government of India) after a senior officer was assigned full-time for this and underwent training for a month at OBIS Directorate at Rutgers University. Affectation to the above Institution also ensures that IndOBIS will have sustained funding support.

Understandably, IO-CoML could facilitate implementation of some Census projects but not all. Some of them, such as satellite tracking of whale sharks (akin to the Census top predator project, TOPP), have been held up for want of governmental approval and funding support. But the IO-CoML has been successful in one respect: it has secured continuity for the Census of Marine Life in India, christened as “CoML-India” and to be implemented by the Ministry of Earth Sciences of the Government of India. In a recent meeting (1 December 2010) on “CoML – what next?” organized by IO-CoML, about 30 senior scientists from India and representatives of regional agencies like WIOMSA (Western Indian Ocean Marine Science Association) and SACEP (South Asia Co-operative Environment Program) met together and identified gap areas in our knowledge and proposed actions needed to be taken up by CoML-India.

## Supporting Information

Appendix S1 List of major taxonomic resources and guides to Indian Ocean marine biota.
(0.06 MB DOCX)
