# Supplementary material for: State of Knowledge of Coastal and Marine Biodiversity of Indian Ocean Countries
Source: PLoS One. 2011 Jan 31;6(1):e14613. doi: 10.1371/journal.pone.0014613 (PMC3031507; doi:10.1371/journal.pone.0014613)
Supplement: Appendix S1 — List of major taxonomic resources and guides to Indian Ocean marine biota. (0.06 MB DOCX) [file pone.0014613.s001.docx]

Appendix S1. List of major taxonomic resources and guides to Indian Ocean marine biota

**Algae**

Desikachary TV (1979) First list of diatoms of the Indian Ocean region. Univ Bot lab Madras 139 p.

Dhargalkar VK, Verlecar XN (2005) The sand Dune flora - a field manual, National Institute of Oceanography Goa 56 p.

Foged N (1975) Some littoral diatoms from the coast of Tanzania. Bibl Phycol 16: 1-128.

Giffen MH (1980) A checklist of marine diatoms from Mahe, Scychelles Islands. Bacillaria 3: 129-159.

Misra JN (1956) A systematic account of some littoral marine diatoms form the West coast of India. J Bombay Nat Hist Soc 53: 537-568.

Oza RM, Zaidi SH (2001) A revised checklist of Indian marine algae. 332 p. Central Salt and Marine Chemicals Research Institute India.

Sournia A (1973) Catalogue des espèces et taxons infra-spécifiques de dinoflagellés marins actuels publies depuis la révision de J. Schiller, I. Dinoflagellés Libres. Nova Hedwigia (Beih.) 48: 1-92.

Sridharan VT (1979) Studies on marine diatoms of the Indian coast. Ph.D. Thesis. Univ Madras. 213 p.

Subrahmanyan R (1946) The diatoms of the Madras coast. Proc Indian Acad Sci 24(B): 87-197.

Subrahmanyan R (1968) The Dinophycea of the Indian Seas. Part I. Genus *Ceratium* Schrank. Mem Mar Biol Ass India 1: 129 p.

Subahmanyan R (1971) The Dinophyceae of the Indian Sea. Part 2. Family Peridiniaceae Schutt emend Lindemann. Mem Mar Biol Ass India 2: 33pp.

Taylor F Jr (1976) Dinoflagellates from the International Indian Ocean Expedition. A report on material collected by the R.V. Anton Bruun 1963- 1964. Bibliotheca bol 132: 1-234.

Venkataraman G (1939) A Systematic account of some South Indian diatoms. Proc Indian Acad Sci 10(B): 293-368.

Wood EJF (1963) Checklist of diatoms recorded from the Indian Ocean. CSIRO Div Fish Oceanogr Rep 36, 315 p.

**Porifera**

Ali MA (1956) Addition to the sponge fauna of Madras. J Madras Univ B26: 289-301.

Anita Mary G, Thomas PA (2000) Identification of marine sponge. *In*: Proc Nat Sem Mar Biodiversity ICAS Pbl 3:39-41.

Burton M (1930) Additions to the sponge fauna of Gulf of Mannar. Ann Mag Nat Hist 30: 665-676.

Burton M (1937) Supplement to the littoral fauna of Krusadai Island. Bull Madras Govt Mus 1(2) pt. 4: 1-58.

Burton M, Rao HS (1932) Report on the shallow water marine sponges in the collection of the Indian Museum. Rec Indian Mus 34(3): 299-356.

Dandy A (1887) The sponge fauna of Madras. A report on a collection of sponges obtained in the neighbourhood of Madras by Edger Thurston. Ann Mag Nat Hist 20(5): 153-164.

Dandy A (1915) Report on the calcareous sponges collected by Mr. James Hornell at Okhamandal in Kattiwar in 1905-1906. Rep Govt Baroda Mar Zool Okhamandal 2: 79-91.

Dandy A (1916) Report on the non-calcareous sponges collected by Mr. James Hornell at Okhamandal in Kattiwar in 1905-1906. Rep. Govt. Baroda Mar. Zool. Okhamandal 2: 96-146.

Dandy A, Burton M (1926) Report on some deep-sea sponges from the Indian Museum collected by RIMS Investigator. I. Hexactincllida and Tetraxonida (Pars). Rec Indian Mus 28: 225-248.

Pattanayak JG (1999) Annotated checklist of marine sponges of the Indian region*.* Mem Queensland Museum 44: 439-455.

Pattanayak JG, Buddadeb M (2001) Distribution of marine sponges (Porifera) in India. Proc Zool Soc Calcutta 54: 73-101.

Thomas PA (1969) Boring sponges of the reefs of Gulf of Mannar and Palk Bay. Proc 1st Int Symp Corals and Coral reefs pp. 333-362.

Thomas PA (1970) On some deep-sea sponges from the Gulf of Mannar with descriptions of three new species. J mar biol Ass India 12: 202- 209.

Thomas PA (1973) The new records of Demospongiae from the Indian Ocean. J mar biol Ass India 14: 443-445.

Thomas PA (1979) Marine Demospongiae of Inhaca Island (Indian Ocean). Bull Royal Afr Cent Belgium 227: 1-45.

Thomas PA (1984) Sponges collected abroad R. V. SKIPJACK from the South East coast of India. J mar biol Ass India 22: 8-20.

**Cnidaria**

Annandale N (1915) Fauna of Chilka Lake. The Coelenterates of the Lake with an account of the Actinaria of brackish water in the Gangetic delta. Mem Ind Mus V: 135-138.

Browne ET (1926) Siphonophorae from the Indian Ocean. Trans Linn Soc Lond (zool.) 19: 55-86.

Daniel R (1991) Siphonophora. In: Animal Resources of India, pp. 35-40.

Daniel R (1985) The fauna of India and the adjacent countries. Coelenterate: Hydrozoa, Siphonophora. Zool Surv India, Kolkata, 440 pp.

Daniel A, Daniel R (1963). A new siphonophore of the genus *Lensia* from the Bay of Bengal. Ann Mag nat Hist Ser 13: 621-623.

Fernando AS, Fernando OJ (2002). A field guide to the common invertebrates of the East Coast of India, Centre of Advanced Study in Marine Biology, Annamalai University, Parangipettai 258 pp.

Leloup E (1934) Siphonophores de Madras (Indes Angalaises) Bull Mus Hist Nat Belg Bruxelles 10: 1-5.

Menon MGK (1931) The Hydromedusae of Madras. Bull Madras Govt Mus (NH) III, 2: 1-32.

Rao DV, Devi K (2002) Studies on the Soft Corals (Octocorallia: Alcyonacea) of Andaman Islands. Bay of Bengal Occ Paper 206 p. Zool Surv India, Calcultta.

Sundararaj B (1927) Siphonophora. Bull Madras Govt Mus (NH) 1: 21-23.

Totton AK (1954) Siphonophora of the Indian Ocean. Discovery Reports **27**: 1-162.

Venkataraman K, Satyanarayanan Ch, Affred, JRB, Wolstenholme, J (2003). Hand book on hard corals of India.266 p. Zool Surv India, Calcultta.

**Chaetognatha**

George PC (1952) A systematic account of the Chaetognatha of the Indian waters with observations on their seasonal fluctuations along the Malabar Coast. Proc Nat Inst Sci India 18: 657-689.

John CC (1933) *Sagitta* of the Madras Coast. Bull Madras Govt Mus Nat Hist Sect 3(4): 1-10.

Rao TSS (1958a) Studies on Chaetognaths of the Indian Sea. II Chaetognaths of the Lawson’s Bay, Waltair. Andhra Univ Mem Oceanogr 2: 137-146.

Rao TSS (1958b) Studies on Chaetognaths of the Indian Seas IV. Distribution in relation to currents. Andhra Univ Mem Oceanogr 2: 164-167.

Rao TSS (1966) Studies on Chaetognaths in the Indian Seas VII. On the occurrence of *Sagitta ferox*  Doncaster and *S. hexaptera* d’Orbigny in the waters off Vishakhapatnam. J Bombay Nat Hist Soc 62: 544-548.

Rao TSS, Ganapati PN (1958) Studies on Chaetognaths in the Indian Seas III. Systematics and distribution in the waters off Vishakhapatnam. Andhra Univ Mem Oceanogr 2: 147-163.

Silas EG, Srinivasan M (1970) Chaetognatha of the Indian Ocean with a key for their identification. Proc Indian Acad Sci 71: 177-192.

Srinivasan M (1979) Taxonomy and Ecology of Chaetognatha of the west coast of India in relation to their role as indicator organisms of water masses. Zool. Surv India Tech Monogr 3: 1-47.

Tardigrada

Rao GC (1971) On two species of marine interstitial Tardigrada from the east coast of India. Proc Indian Acad Sci LXXIII (2): 53-57.

Rao GC (1972) Ocurrence of the interstitial Tardigrada *Parastyogarctus higginsi* Renaud-Debyser in the intertidal sands on Andaman Islands. Curr Sci 41: 845-846.

Rao GC (1991) Meiofauna, In: The Fauna of Lakshadweep. Zool Surv India, Kolkata: 41-135.

**Platyhelminthes**

Fauvel P (1953) The fauna of India including, Pakistan, Ceylon, Burma and Malay. Annelida, Polychaeta, XII 507 pp. Zool Surv India, Calcutta

Hyman L (1951) The Invertebrates: Platyhelminthes and Rhynchocoela.Vol.II, McGraw Hill Book Co. Inc.

Mehra HR (1980) Fauna of India and the adjacent countries, Platyhelminthes Vol. I. Trematoda: Digenea. Zool.Surv.India, Calcutta.

Southwell T (1930) Fauna of British India including Ceylon and Burma¸1-391.

Srivastava CB (1992) The Fauna of Indian and adjacent countries platyhelminthes, Vol.1, (Supplements) Trematode: Digenea 1-163. Zool Surv India, Calcutta.

Yamaguti S (1959) Systema helminthun-II, Interscience New York, 1-860.

Yamaguti S (1971) Synopsis of digentic trematodes of vertebrates, Vol I & II, Keigaku Publishing Co, Tokyo 1074 p.

Echiura

Haldar BP (1978) Notes on some Echiura from the east coast of India. Bull Zool Surv India 1(3): 315-326.

Haldar BP (1995a) Echiura and Sipuncula. Part 1: Fauna of Chilka Lake. Zool Surv India Wetland Ecosystem Series 476-479.

Haldar BP (1995b) Echiura and Sipuncula. Part 2. Hugli-Matla Estuary. Zool. Surv. India, Estuarine Ecosystem Series 31-39.

Haldar BP, Dattagupta AK (1991) Echiura. In. State Fauna Series 2: Fauna of Lakshadweep: 185-197.

Haldar BP (1998) Faunal diversity in India: Echiura (in) Fauna: Diversity in India (Editors Alfred JRB, Das AK, Sanyal AK) 120-122.

**Sipuncula**

Haldar BP (1991) Sipunculans of the Indian Coast. Mem Zool Surv India 17(4): 164 pp.

**Annelida: Polychaeta**

De Silva PHDH (1961) Contribution to the knowledge of the polychaete fauna of Ceylon. Bull Nat Mus Ceylon 29(2): 164-194.

Fauvel P (1953) Annelida Polychaeta. The Fauna of India Including Pakistan, Ceylon, Burma and Malaya. The Indian Press Ltd, Allahabad, 507 p.

Fauvel P (1930) Annelida polychaeta of the Madras Museum. Bull Madras Govt Mus (N.S) Supplement 1(1):1-8.

Fauvel P (1932) Annelida Polycheata of the Indian museum, Culcutta. Mem Indian Mus 12:1-262.

Fauvel P (1934a) Sur quelques Syllidiens du Japon. Annot Zool Japonenses XIV No.3: 301-315.

Fauvel P (1934b) Annelides Polychaetes de Rovigno-d’Istria. Thalassia 1(7) : 1-78.

Fishelson L, Rullier F (1969) Quelques annélides polychètes de la Mer Rouge. Israel J Zool 18: 49-117.

Gravier C (1901) Contribution a l’étude des annélides polychètes de la Mer Rouge. Nouv Arch Mus Hist Nat Paris (ser.4) 3: 147-268.

Hartman O (1974a) Polychaetous annelids of the Indian Ocean including an account of species collected by members of the international Indian Ocean Expeditions, 1963-64 and a catalogue and bibliography of the species from India. Part 1. J mar biol Ass India 16: 191-252.

Hartman O (1974b) Polychaetous annelids of the Indian Ocean including an account of species collected by members of the international Indian Ocean Expeditions, 1963-64 and a catalogue and bibliography of the species from India. Part 2. J mar biol Ass India 16: 609-644.

Sanjeeva Raj PJ (1976) Review of fish-leaches of the Indian Ocean. J mar biol Ass India 16: 381-397.

Southern R (1921) Polychaeta of the Chilka Lake and also the fresh and brackish waters in other parts of India. Mem Indian Mus 5: 563-659.

**Bryozoa**

Annandale N (1908) The fauna of Brackish ponds at Port Canning, Lower Bengal VII. Description of a new genus *Entroprocta*. Rec Ind Mus 2: 24-32.

Annandale N (1910) Fauna of British India. Vol. I. Freshwater sponges, Coelentrates and Polyzoa.

Harmer SF (1915) The polyzoa of the Siboga expedition. I-IV. Enteoprocta, Ctenostomata, Cyclostomata. Siboga Exped 28: 1-1147.

Menon NR (1967) Studies on the polyzoa of the southwest coast of India. Ph.D. Thesis Univ Kerala 548 p.

Rao KS (1991) *Entroprocta.* In: Animal Resources of India. State of the Art. 549-550. Zool Surv of India.

Robertson A (1921) Report on a collection of Bryozoa from the Bay of Bengal and other eastern seas. Rec Indian Mus. 22: 33-65.

Thornely LR (1907) Report on the marine polyzoa in the collection of the Indian Museum. Rec Indian Mus 1: 179-196.

**Mollusca**

Adam W (1939) The Cephalopoda in the Indian Museum, Calcutta. Rec Indian Mus 41: 61-110.

Adam W, Rees WJ (1966) A review of the Cephalopod family Sepidae. Sci Rep John Murray Exped 1933-34 11: 1-165.

Crichton MD (1940) Marine shells of Madras. J Conch London, 21: 193-212.

Crichton, MD (1941) Marine Shells of Madras. J Bombay Nat Hist Soc, 42(2): 323-341.

Fillippova JA (1968) New Data on the Cephalopoda of the Indian Ocean. Proc Symp Mollusca Part-I 257-264. Cochin.

Hoyle WE (1886) Report on the Cephalopoda collected by H. M. S. Challenger during the years 1873-1876. Rep Sci Res Voy Challenger, Zool. **16**: I-VI, 1-246, pls. 1-33.

Goodrich ES (1896) Report on a collection of Cephalopoda from the Calcutta Museum. Trans Linn Soc Lond **7**(1): 1-24.

Gravely FH (1942) Shells and other animal remains found on the Madras Beach II. Snails etc. (Mollusca: Gastropoda). Bull Madras Govt Mus NS (Nat Hist) 5(2): 1-104.

Hornell J (1922) The common molluscs of South India. Madras Fish Bull 14: 97-215.

Hornell J (1951) The study of Indian Molluscs. J Bombay Nat Hist Soc 48: 303-374.

Satyamoorthy ST (1952) The Mollusca of Krusadai Island (in the Gulf of Mannar) Bull Madras Govt Mus New ser Nat Hist Sect 1(2) pt. 6: 1-258.

Satyamoorthy ST (1956) Mollusca of Krusadai Island (in the Gulf of Mannar) II. Scaphopoda, Pelecypoda and Cephalaopod. Bull Madras Govt Mus New ser Nat Hist Sect 1(2) pt. 7: 202.

**Subba Rao NV, Dey, A (2000)** Catalogue of Marine molluscs of Andaman and Nicobar Islands. Rec Zool Surv India Occ Paper. 187: 1-323.

Ray HC (1948) On a collection of mollusca from the Coromandel coasts of India. Rec Indian Mus 46: 87-121.

Winckworth R (1936) Marine mollusca from South India and Ceylon. Proc Malac Soc London 22: 16-23.

**Crustacea**

Adams A, White A (1848) Crustacea. In: Adams, The zoology of the Voyage of H. M. S. Samarang, 1843-1846, London, pp. I-VIII 1-66 pls.1-13.

Alcock A (1895) Materials for a Carcinological Fauna of India No. 1. The Brachyura Oxyrhncha. J Asiatic Soc Bengal 64(2): 158-291 pls.3-5.

Alcock A (1896) Materials for a Carcinological Fauna of India No. 2. Brachyura Oxystomata. J Asiatic Soc Bengal 64(2): 134-296, pls.6-8.

Alcock A (1898) Materials for a Carcinological Fauna of India No. 3. Brachyura Cyclometopa, part- I. The family Xanthidae. J Asiatic Soc Bengal 67(2): 67-283.

Alcock A (1899) Materials for a Carcinological Fauna of India No. 5. Brachyura Primigenia or Dromiacea. J Asiatic Soc Bengal 68(3): 123-169.

Alcock A (1900) Materials for a Carcinological Fauna of India No. 6. Brachyura Catametopa or Graspsoidea. J Asiatic Soc Bengal 69(2): 123-169.

Alcock A (1901) A Descriptive catalogue of the Indian Deep-Sea Crustacea, Decapoda, Macrura and Anomura in the Indian Museum, being a revised account of the Deep-Sea Species collected by the Royal Marine Survey Ship “Investigator”. Calcutta, India, 286 pp. Zool. Surv. India.

Alcock A (1901) Catalogue of the Indian Decapod: Crustacea in the collection of the Indian Museum, Part- I. Brachyura. Fasc I. Introduction and Dromides or Dromiacea (Brachyura: Primigenia) Calcutta, pp. 1-80, pls. 1-8. Zool. Surv. India

Alcock A (1905) Catalogue of the Indian Decapod Crustacea in the collection of the Indian museum. Part. II. Anomura. Trustees of the Indian Museum Culcutta, p.197.

Annandale N (1909) An account of the Indian *Cirrepedia pedunculata*. Pt. I, Family: Lepadidae (*Sensu stricto*). Mem Ind Mus 2: 61-137.

Annandale N (1910) The Indian Barnacles of the sub-genus *Smilium* with remarks on the classification of the genus *Scapellum*. Rec Indian Mus 5:145-155.

Annandale N (1913) The Indian Barnacles of the sub-genus *Scapellum*. Rec Indian Mus 9: 227-236.

Bernard KH (1935) Report on some Amphipoda, Isopoda, and Tanaidacea in the collection of the Indian Museum. Rec Ind Mus 37: 279-319.

Bernard KH (1937) Amphipoda. The John Murray Expedition, 1933-34. Scientific Reports Brit Mus (Nat Hist) 4(6): 131-201.

Chopra BN (1933a). Further notes on Crustacea Decapoda in the Indian Museum- III. On the Decapod: Crustacea coll. by the Bengal Pilot Service off the Mouth of the River Hooghly. Brachygnatha (Oxyrhyncha and Brachyrhyncha). Rec Indian Mus 37: 463-514.

Chopra BN (1933b). Further notes on Crustacea Decapoda in the Indian Museum- III. On the Decapod: Crustacea coll. by the Bengal Pilot Service off the Mouth of the River Hooghly. Dromiacea and Oxystomata Rec Indian Mus 35: 25-52.

Chopra BN (1935) Further notes on Crustacea Decapoda in the Indian Museum- III. On the Decapod: Crustacea coll. by the Bengal Pilot Service off the Mouth of the River Hooghly. Brachygnatha (Oxyrhyncha and Brachyrhyncha). Rec Indian Mus 37: 463-514.

Chopra BN, Das KN (1937) Further notes on Crustacea Decapoda in the Indian Museum- IX on three collections of Crabs from Tavoy and Mergui Archipelago. Rec Indian Mus 39: 377-434.

Fernando SA (2006). Monograph of Indian barnacle. OASTC Marine Benthos – 02 Ocean Science and technology cell, CUSAT, Kochi.

Sewell RBS (1932) The Copepoda of Indian Seas. Calanoida. Mem Indian Mus Calcutta 10:223–407.

Kasturirangan LR (1963) A key for the identification of the more common planktonic Copepoda of Indian coastal waters. Indian National Commission on Oceanic Research, Council for Scientific and Industrial Research. New Delhi, India. 2:1–87.

Ajmal Khan S, Natarajan, R (1984). Hermit crabs of Porto Novo Coast. Rec Zool Survey of India Misc Publ Occa Paper 67:1–25.

Tattersall WM (1922) The Percy Sladen Trust Expeditions to the Abrolhos Islands (Indian Ocean). Amphipoda and Isopoda. J Linn Soc Lond Zool 25: 1-19.

Walker AO (1904) Report on the Amphipoda. Report to the Government of Ceylon on the Pearl Oyster Fisheries in the Gulf of Mannar. Roy Soc London Pt. II, Supplementary Report17: 229-300.

Walker AO (1905) Marine Crustaceans, XVI, Amphipoda. The Fauna and Geography of the Maldive and Laccadive Archipelagoes, 2, Supplement-I, 923-932, pl.88.

Walker AO (1909) Amphipoda Hyperiidea of the 'Sea-lark' Expedition to the Indian Ocean. The Percy Sladen Trust Expedition to the Indian ocean in 1905. Trans Linn Soc London ser.2 Zool 13(1): 49-55.

**Echinodermata**

Bell FJ (1884) “Echinodermata”, Report on the Zoological Collections made in the Indo-Pacific Ocean during the Voyage of HMS Albert, 1881-1882, British Museum, London, 1884, pp. 117-177.

Bell FJ (1888) Report on a collection of Echinoderms made at Tuticorin, Madras, by Mr. Edgar Thurston, CMZS, Superintent, Government of Central Museum, Madras. Proc Zool Soc London, 383-389.

Bell FJ (1887) The echinoderm fauna of the island of Ceylon. Scient Trans R Phil Soc 3: 643-658.

Bell FJ (1889) Additions to the Echinoderms of the Bay of Bengal. Proc Zool Soc Lond 1889: 6-7.

Bell FJ (1902) The Actinogonidiate Echinoderms. Of the Maldive and Laccadive Islands. In Gariidiner. J.S. The Fauna and Geography of the Maldive and Laccadive Archipelagoes. Cambridge, 1 (3): 223-233.

Cherbonnier G, Guike, A (1978). Echinoderma ophiurides. Fauna de Madagascar. CNRS, Paris 48: 272pp.

Clark AH (1912) The Crinoids of the Indian Ocean, 325 pp.

Clark AH (1912) Crinoidea. Echinoderma of the Indian Museum. Calcutta, 1-325.

Clark AH (1932) On a collection of crinoids from the Indian Ocean and the Bay of Bengal. Rec Indian Mus 34(4): 551-566.

Daniel A, Haldar BP (1974) Holothuroidea of the Indian Ocean with remarks on their distribution. J Mar biol Ass India 16: 412-436.

James DB (1969) Catalogue of Echinoderms in the refernce collection of the Central Marine Fisheries Research Institute, Madapam. Bull Cent Mar Fish Res Inst 7: 51- 62.

Koehler R (1910) Asteris du Musee de Calcutta: II Les Asteries Littorals Echinoderma of the Indian Museum. Part VI.Indian Museum. Calcutta, 192pp.

Koehler R (1914) Echinides du Musée Indien à Calcutta. I. Spatangides. Echinoderma of the Indian Museum.Part VIII. Echinodea(1). Indian Museum. Calcullta, 258pp.

Koehler R (1922) Echnides du Musee Indian a Calcutta. II. Chypeaterides st Cassidulides. Echinoderma of the Indian Museum, Part IX. Echinoidea (II).Indian Museum, Calcutta, 161pp.

Koehler R (1927) Echinides du Musee Indian a Calcutta, III. Echinides Reguliers. Echinoderma of the Indian Museum. Part IX. Echinodea (III). Indian Museum, Calcutta, 158pp.

Koehler R, Vaney, CV (1905) Holothuries necuellies Part 1’ Investigator dans I’ Ocean Indien. I.Les holothuries de mer Profonde. Echinoderma of the Indian Museum. Indian Museum, Calcutta, Part III. 123 & iipp.

Koehler R, Vaney CV (1908) Holothuries necuellies Part 1' Investigator dans I’ Ocean Indien. Les holothuries Littorales. Echinoderma of the Indian Museum, Part IV Indian Museum, Calcutta, 54pp.

Marsh LM, Priu ARG (1991) Indian Ocean Echinoderms collected during the Sindbad Voyage (1980-81) : 2, Asteroidea. Bull Br Mus Nat Hist (zool) 57(1): 61-70.

Marshall JI, Rowe FEW (1981) The crinoids of Madagascar. Bull Mus Nat Hist Nat Paris Ser 3, Section A, no.2: 379- 413.

Satyamurthi STn (1976) The Echinoderms in the collection of the Madras Government Museum. Bull Madras Govt Mus New Ser Nat Hist VII(3): 279 pp.

Theel H (1886). Holothuridea II, Report on Scientific Research, HMS Challenger, Zoology XIV, 290 pp.

**Hemichordata**

Ramunni Menon K (1994) Enteropneusta from Madras. Quart J micr Sci 47: 123-131.

Singh BN, Choudhury A (1992) Hemichordata. Fauna of West Bengal, State Fauna Series 3. Rec Zool Surv India 2: 243-438.

Dandapani P (1998) Faunal diversity in India: Hemichordata, (in) Faunal Diversity in India, i-viii, 406-409. Zool Surv India.

Protochordata

Azaria J (1965) Studies on the Cephalochordates of Madras Coast. I. Taxonomic study. J Mar biol Ass India 7(2): 348-363.

Dandapani P (1996) Pelagic tunicate from the seas around Andaman and Nicobar Islands. Proc Second Workshop Sci Results *FORV Sagar Sampada*, 217-227.

Dandapani P (1998) Faunal diversity in India: Protochordata, (in) Faunal Diversity in India, i-viii, 412-415. Zool Surv India.

Sewell RBS (1926) The Salps of the Indian Seas. Rec Ind Mus **28:** 65-126.

Meenakshi VK (2000) Ecteinascidia venui sp. nov., a colonial ascidian (Perophoridae) from Tuticorin, southeast coast of India. Indian J Mar Sci 29: 83-85.

Meenakshi VK, Senthamarai, S (2004) First report of a simple ascidian – *Phallusia arabica* Savigny, from Tuticorin coast of India. J mar biol Ass India 46(1): 104–107.

Meenakshi VK, Senthamarai S (2006) First report on two species of ascidians to represent the genus *Botryllus* Gaertner 1774 from Indian waters. J mar biol Ass India 48(1): 100-102.

Meenakshi VK, Senthamarai S (2006) First report of a simple ascidian – *Pyura spinosa* (Quoy and Gaimard, 1834) from Tuticorin coast of India. J mar biol Ass India 48(1):103-104.

**Vertebrata (Pisces)**

Allen GR, Steena RC (1987) Reef fishes of the Indian Ocean. T.F.H. Publications, Neptune City.

Barman RP, Mishra SS (2005) Studies on the reef dwelling fishes of India, Parrot fishes (Family: Scadidae) Zool Surv India, Kolkata, 63 pp.

Barman RP, Mishra SS (2009). A Pictorial guide to the fishes of the Family Nemipteridae of India, Zool Surv India, Kolkata, 50 pp.

Day F (1875-78) The fishes of India, being Natural History of the fishes known to inhabit the seas and fresh water of India, Burma and Ceylon. Text and Plates, London.

Day F (1889) The fauna of British India, including Ceylon and Burma, Fishes. Vol. I and II. Taylor and Francis, London.

Fischer W, Bianchi G (1984) FAO Species identification sheets for fishery purposes, Eastern Indian Ocean (Fishing area 51) volumes, 1-5; Food and Agriculture Organization of the United Nations, Rome.

Fischer W, Whitehead PJP (1974) FAO species identification sheets for fishery purposes, Eastern Indian Ocean (Fishery area 57) and Western Central Pacific (Fishing area 71). Vol.1-4. Food and Agricultural Organization of the United Nations, Rome.

Jones S, Kumaran M (1980) Fishes of the Laccadive Archipelago. The nature conservation and aquatic science service, Trivandrum, 760 p.

Mohsin AKM, Ambak MA (1996) Marine fishes and fisheries of Malaysia and neighbouring countries. University Pertanian Malaysia Press, 744 p.

Nelson JS (1976) Fishes of the World. John Wiley and sons, New York, USA.

Talwar PK, Kacker RK (1984) Commercial Sea fishes of India, Zool. Surv. India, 997p.

Rajan PT (2003) A field guide to the Marine food fishes of Andaman and Nicobar islands: 1-260. Zool Surv India.

Weber M, de Beaufort LF (1936) The fishes of the Indo-Australian Archipelago. Vols. 1-10; A.J. Reprints Agency, 24 B/5, Karolbagh, New Delhi.

|  |  |
| --- | --- |
|  |  |
|  |  |

**Other vertebrates**

Ahmed S (1975) Sea snakes of the Indian Ocean in the collection of Zoological Survey of India together with remarks on the geographical distribution of the all Indian species. J Mar biol Ass India 17(1): 73-81.

Agrawal VC, Alfred JRB (1999) Handbook on Whales, Dolphins and Dugong from Indian Seas, i-iv, 1-150. Zool Surv India

Balachandran S (1996) Shore birds of the Marine National Park in the Gulf of Mannar, Tamil Nadu. J Bombay Nat His Soc 92: 303-313.

Daniel JC (1983) The book of Indian Reptiles. Bombay Nat Hist Soc Bombay India 141 p.

Dhargalkar VK, Verlecar XN (2005) Marine Mammals, Turtles and Crocodiles- a field manual. National Institute of Oceanography, Goa, 60 pp.

Dhargalkar VK, Verlecar XN (2005) Marine and Coastal Birds- a field manual. National Institute of Oceanography, Goa, 28 pp.

Haile, N.S. 1958. The snakes of Borneo, with a key to the species. Sarawak Museum Journal., 8, 743-771.

James PSBR, Lal Mohan RS (1987) The marine mammals of India. Mar Fish Infor Serv 71:1-19.

Karthikeyan, R. and T. Balasubramanian. 2007. Species diversity of the sea snake (Hydrophiidae) distributed in the Coromandel Coast (East Coast of India). Internatl. J. Zool. Res., 3,107-131.

Murthy TSN (1977) On sea snakes occurring in Madras waters. J mar biol Ass India 19(1): 68-72.

Murthy TSN (1999) Marine Reptiles of India. An overview to contribution of herpetology. Herpetologica. 58(2): 35-38.

Natarajan R, Rajaguru A (1985) Small cetaceans. Mar Mam Sci 1:89.

Ravichandran MS, Murthy TSN (1998) Faunal diversity in India: Reptilia. Zool Surv India, Calcutta, pp. 445-447.

Smith MA (1933) The fauna of British India including Ceylon and Burma. Reptilia and Amphibia Vol. **I**, Loricata, Testudines, Taylor and Francis, London;, **xxviii** + 185pp.

Smith MA (1935) The fauna of British India including Ceylon and Burma. Reptilia and Amphibia Vol. - **II**, Sauria, Taylor and Francis, London **ix** +440pp.

Smith MA (1943) The fauna of British India including the whole Indo-Chinese sub-region. Reptilia and Amphibia Vol - **III,** Serpentes, Taylor and Francis, London, **vii** +583 pp.

Tikader BK, Sharma RC (1985) Handbook of Indian Testudines. Zool Surv India Calcutta **xii**+156 pp.

Tikader BK, Daniel A, Subba Rao NV (1980) Sea shore animals of Andaman and Nicobar islands. Zool Surv India.
